# Reductive Photodegradation of 4,4′-Isopropylidenebis(2,6-dibromophenol) on Fe_3_O_4_ Surface

**DOI:** 10.3390/ma16124380

**Published:** 2023-06-14

**Authors:** Joanna Kisała, Bogdan Stefan Vasile, Anton Ficai, Denisa Ficai, Renata Wojnarowska-Nowak, Tomasz Szreder

**Affiliations:** 1 Institute of Biology, College of Natural Sciences, University of Rzeszow, Pigonia 1 Str., 35-310 Rzeszow, Poland; 2 National Research Center for Food Safety, University Politehnica of Bucharest, Splaiul Independentei 313, 060042 Bucharest, Romania; bogdan.vasile@upb.ro; 3 Faculty of Chemical Engineering and Biotechnologies, University Politehnica of Bucharest, 1-7 Gh. Polizu St., 060042 Bucharest, Romania; anton.ficai@upb.ro (A.F.); denisa.ficai@upb.ro (D.F.); 4 Institute of Materials Science, College of Natural Sciences, University of Rzeszow, Pigonia 1 Str., 35-959 Rzeszow, Poland; rwojnarowska@ur.edu.pl; 5 Institute of Nuclear Chemistry and Technology, Dorodna 16, 03-195 Warsaw, Poland; t.szreder@ichtj.waw.pl

**Keywords:** magnetite, photocatalysis, Advanced Oxidation or Reduction Processes (AOPs/ARPs), TBBPA

## Abstract

Background: Advanced Oxidation Processes (AOPs) are the water treatment techniques that are commonly used forthe decomposition of the non-biodegradable organic pollutants. However, some pollutants are electron deficient and thus resistant to attack by reactive oxygen species (e.g., polyhalogenated compounds) but they may be degraded under reductive conditions. Therefore, reductive methods are alternative or supplementary methods to the well-known oxidative degradation ones. Methods: In this paper, the degradation of 4,4′-isopropylidenebis(2,6-dibromophenol) (TBBPA, tetrabromobisphenol A) using two Fe_3_O_4_ magnetic photocatalyst (F1 and F2) is presented. The morphological, structural and surface properties of catalysts were studied. Their catalytic efficiency was evaluated based on reactions under reductive and oxidative conditions. Quantum chemical calculations were used to analyse early steps of degradation mechanism. Results: The studied photocatalytic degradation reactions undergo pseudo-first order kinetics. The photocatalytic reduction process follows the Eley-Rideal mechanism rather than the commonly used Langmuir-Hinshelwood mechanism. Conclusions: The study confirms that both magnetic photocatalyst are effective and assure reductive degradation of TBBPA.

## 1. Introduction

Water scarcity limits access to clean and safe water for many people on Earth. While the amount of freshwater on the planet remains constant over time, water availability is decreasing. Water deficiency currently affects over 40% of the world’s population [1]. The development of industry does not improve the situation due to the production of large amounts of organic contaminated wastewater. Many of the compounds contained in these wastewaters are classified as persistent in the environment. Considering the shortcomings in access to water, it is necessary to develop effective methods of wastewater treatment.

Advanced Oxidation Processes (AOPs) are treatment technologies designed to degrade recalcitrant organic compounds in wastewater using the reactive oxygen species (ROS), especially hydroxyl radicals (^•^OH). These technologies have been developed as a solution to remove emerging contaminants, especially pharmaceuticals, personal care products, and pesticides. AOPs cover homogeneous and heterogeneous photocatalysis, the Fenton process, Fenton-like processes, ozonation, the use of ultraviolet irradiation, ultrasound, microwaves, gamma irradiation, electrochemical processes, and wet oxidation processes [2,3]. AOPs break down non-biodegradable organic compounds into easier biodegradable intermediates. Some of the organic pollutants are electron-deficient and thus stable against reactive oxygen species, e.g., polyhalogenated compounds. Therefore, reductive methods can be used alternatively when the oxidative degradation is not suitable.

Advanced Reduction Processes (ARP) are water treatment techniques where chemical contaminants undergo reductive conversion. ARPs are based on the production of highly reducing species (by combining reducing agents and activation methods), such as the aqueous electron (e_aq_) and the hydrogen atom (^•^H) [4]. The e_aq_ is the most powerful reducing agent with a reducing potential of −2.9 V. Compared with ^•^OH radical, e_aq_ demonstrated a higher reactivity toward many organic compounds, especially halogenated organic compounds [5,6]. Koester and Asmus [7] found that hexafluorobenzene reacts with e_aq_ more efficiently than with the ^•^OH. The ‘hydrated electron’ is the primary reducing radical formed by water radiolysis [8]. Some degradation methods break down contaminants by oxidising and reducing radicals, so they should be called advanced oxidation-reduction processes (AORPs), but sometimes they are called AOPs or ARPs, depending on how the target compound is degraded (oxidatively or reductively). The reaction mechanism depends on the photocatalyst material, substrate, and reaction medium.

Photocatalysis is an advanced technology for the removal of organic pollutants from aqueous systems [9,10]. So far, many studies of photocatalytic reactions promoted by aqueous suspensions of metal oxides have been carried out [11,12,13]. Photoexcitation of catalysts produces electrons (e^−^) in the conduction band and holes (h^+^) in the valence band. Such a hole-electron pair is very unstable and can recombined or taken part in oxidation and reduction reactions on the surface of the catalyst [14].

Magnetite is a naturally occurring iron oxide with a mixed valence made of Fe^3+^ and Fe^2+^ [15]. Magnetite was recognized as a potentially important catalyst for reductive degradation of environmental pollutants, such as halogenated organic compounds. This is due to the ubiquity of magnetite in the environment and its unique structure which enables the electron hopping between Fe sites. In this paper, we present a degradation of 4,4′-isopropylidenebis(2,6-dibromophenol) (tetrabromobisphelol A, TBBPA) using the two magnetite photocatalysts. In particular, the reductive process was investigated and compared with the effectiveness of the oxidative experiment.

## 2. Materials and Methods

### 2.1. Materials

4,4′-isopropylidenebis(2,6-dibromophenol) (tetrabromobisphenol A, TBBPA) (97%) was purchased from Alfa Aesar (Haverhill, MA, USA), tert-butanol (*t*-BuOH) (Honeywell/Riedel-de-Haen, Seelze, Germany). Magnetites were obtained from Sigma-Aldrich: Fe_3_O_4_ (F1), characterise by particle diameter of ~5 µm, a density of 4.8–5.1 g cm^−3^ and 95% trace metals while, the second samples of magnetite nanopowder (F2) was characterised by a diameter of less than 50 nm, a density of 4.8–5.1 g cm^−3^ and >98% trace metals. All chemicals used in these experiments were of analytical grade and were used without additional purification.

### 2.2. Photocatalysts’ Characterisation

The point of zero charge (PZC) of magnetites was appointed following Kocharova et al. [16]. For the determination of pH_pzc_, eleven solutions of 0.1 mol/dm^3^ NaCl with various pH were prepared. pH were adjusted in range 2–12 with solutions of NaOH and HCl. Then 10 mg of magnetic photocatalyst was dispersed in each solution. The dispersions were stirred continuously at room temperature, for 3 h at 240 rpm to reach the steady state. After this period, the final pH of the two solutions was determined using a CPC 411 multimeter (Elmetron, Zabrze, Poland). The results were plotted as function pH_final_ = f(pH_initial_). The PZC value was obtained at the intersection of pH_final_ = f(pH_initial_) and y = x on the graph.

The morphology of the magnetic photocatalysts was evaluated with a field emission scanning electron microscope (FESEM; Zeiss Ultra 55, Oberkochen, Germany). The TEM images (bright field (BFTEM)), high resolution (HRTEM) and selected area diffraction patterns (SAED) were obtained using a high resolution Titan Themis transmission electron microscope from Thermo Fisher Scientific (Waltham, MA, USA). The microscope was operated in transmission mode at a voltage of 200 kV [17]. The samples preparation technique was described by Vasile et al. [18] and comprised placing a small amount of powder dispersed into ethanol onto a 400 mesh holey carbon-coated film copper grid.

Raman spectra were recorded at room temperature using InVia Micro Raman Renishaw spectrometer (Renishaw, Wotton under Edge, Gloucestershire, UK) coupled with a Leica DM 2500 M microscope (Leica Microsystems GmbH, Wetzlar, Germany), using two lasers as an excitation source (633 nm and 785 nm). The spectra were recorded with an exposure time of 120 s by co-adding 3 scans in eight places on the sample. The laser output power on the level of 0.05 mW for 785 nm and 0.15 mW for 633 nm was used to avoid the thermal effect and not alter the sample chemically and structurally.

Cu-Kα powder diffractometer (D8 Advance, Bruker, Ettlingen, Germany) operated at 40 kV and 36 mA (λ = 0.154056 nm) was used to evaluate the crystalline phases from the samples.

The optical characterisation of the catalysts was performed by an Agilent Technologies Cary Series UV-Vis-NIR spectrophotometer (Agilent Technologies, Inc., Santa Clara, CA, USA) in the wavelength range of 190 to 800 nm.

### 2.3. Photocatalytic Degradation of TBBPA

The photocatalytic activity of F1 and F2 was assessed by monitoring the degradation of TBBPA. For this purpose, 250 cm^3^ of the TBBPA solution (concentration 2 × 10^−3^–15 × 10^−3^ mol dm^−3^) containing 300 × 10^−3^ mol dm^−3^ *t*-BuOH (hydroxyl radical scavenger, only in experiments favouring reductive decomposition) was transferred to the reactor. The pH of the solution was adjusted to 10 ± 0.1 using a 0.1 mol dm^−3^ NaOH solution. The catalyst was dispersed in constantly stirred reaction mixture, at a concentration of 0.150 g dm^−3^. The suspension was maintained for 30 min, in the dark, for reaching adsorption/desorption equilibrium under argon conditions (reductive experiment) or open to air (oxidative experiment). Photocatalytic degradation has been carried out using a Heraeus LRS2 glass photoreactor (Heraeus, Hanau, Germany) using a TQ150 excimer lamp (150 Watt, water-cooled to 25 °C, 47 Watt light energy flux of power density 4.7 mW cm^−2^ measured by the digital lux meter Peak Tech) which was immersed in the suspension. The process was carried out for 120 min. During the experiment, 2 cm^3^ samples were removed from the reactor at regular time intervals (at every 2 min in the first 10 min and after this at every ten minutes). The concentrations of the organic compound were assessed using the HPLC technique. Prior the injection, the solutions were filtered through a 0.22 µm syringe filter.

### 2.4. Analytical Methods

Changes in TBBPA concentration were determined by high performance liquid chromatography (HPLC, Shimadzu, Kyoto, Japan) equipped with a UV detector (SPD-10 AV) and a C18 column (Knauer 250 mm × 4.6 mm with precolumn, Eurospher II, 100-5 C18 H). Analysis conditions: mobile phase—70% acetonitrile and 30% water, flow rate: 1.0 cm^3^ min^−1^, injection volume: 20 × 10^−3^ cm^3^, absorbance detection: 310 nm. For calibration, nine standardised TBBPA solutions with concentration levels ranging from 2.0 × 10^−5^ to 1 × 10^−6^ mol dm^−3^ were used.

The concentration of bromide ions was assessed using a potentiometric technique using a bromide-ion-selective electrode (EBr-01, Hydromet, Gliwice, Poland) with a silver chloride reference electrode (RL-100, Hydromet, Gliwice, Poland) and a multimeter (CPC 411, Elmetron, Zabrze, Poland). For the quantification of the bromide ions, seven standardised bromide solutions with concentration levels ranging from 10^−5^ to 10^−2^ mol·dm^−3^ were used.

### 2.5. The Quantum-Chemical Calculations

Calculation was carried out with Gaussian 09 W [19] program package using Hartree-Fock method (HF) [20] and range separated hybrid functional (LC-ωPBE) [18] theory levels. These methods were previously verified for investigation of dissociation of simple molecular systems [21,22]. Geometry optimizations and electronic calculations for all the species were carried out in the gas phase. No imaginary frequencies were found for optimised structures. Visualisation of the quantum-chemical calculations was carried out by Chemcraft 1.8 (evaluation version).

## 3. Results and Discussion

### 3.1. Characterisation of Catalysts

**Figure 1:**
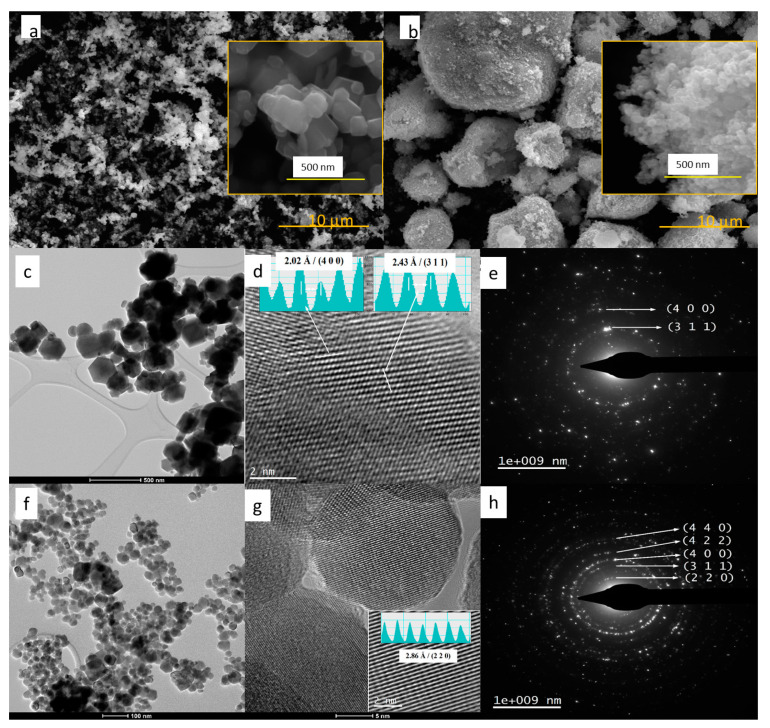
SEM images of F1 (**a**) and F2 (**b**) catalyst; Bright Field TEM images obtained on sample F1 (**c**) and F2 (**f**); HRTEM images obtained on sample F1 (**d**) and F2 (**g**); SAED pattern obtained on sample F1 (**e**) and F2 (**h**).

The morphology of Fe_3_O_4_ catalysts is shown in Figure 1a,b. Nanocrystal agglomerates with sizes in range 100–400 nm (Figure 1a) and 25–100 nm (Figure 1b) are observed for F1 and F2 catalysts, respectively. The BFTEM images of F1 (Figure 1c), presented that the sample is highly inhomogeneous in terms of the shape and size of the structures (50–250 nm). The catalyst is composed of polyhedral, hexagonal, and spherical-shaped particles. HRTEM image reveals highly crystallised particles, with orientations corresponding to the Miller indices (4 0 0) and (3 1 1) of 2.02 Å and 2.43 Å, respectively (Figure 1d). The SAED pattern of the F1 catalyst reveals that the resulting phase is Fe_3_O_4_ as it corresponds to ICDD reference data 00-026-1136 (Figure 1e). The obtained pattern also confirms the large size of the particles, as the patterns do not consist of clearly concentric circles, but rather of dots scattered across the image. The BFTEM image of F2 (Figure 1f), showed that the sample is homogeneous in terms of the shape and size of the structures, with particles of approximately 25 nm. There are also larger particles not exceeding 100 nm. The sample is mostly composed of spherical particles with small fraction polyhedral one. The HRTEM image (Figure 1g insert) reveals a highly crystallised particle, in which one can identify crystal orientations corresponding to the Miller indices (4 0 0) and (2 2 0) of 2.02 Å and 2.86 Å, respectively. The particles are monocrystalline, and their SAED pattern (Figure 1h) reveals that the resulting phase is Fe3O4 as it corresponds to ICDD reference data 00-026-1136. The SAED patterns clearly comprise concentric circles, which indicates the nano size of F2 particles.

**Figure 2:**
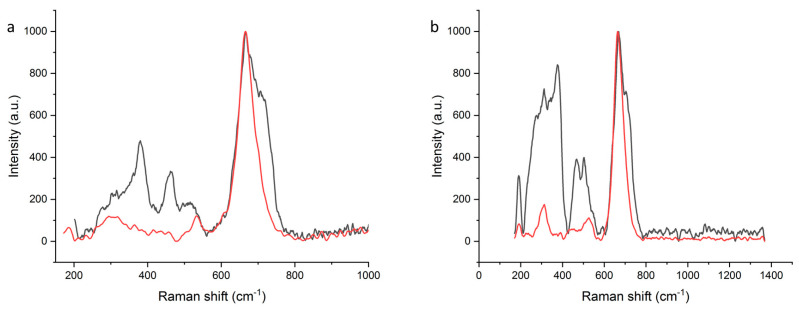
Raman spectra of F1 (red line) and F2 (black line) obtained when excited with a wave of length 633 nm (**a**) and 785 nm (**b**).

Raman spectroscopy is very useful for differentiating between spinel ferrites due to their phonon modes. Collected Raman spectrum of the F1 sample (Figure 2) indicates the magnetite structure. The most intense signal is observed at 667 cm^−1^, which can be assigned to an A_1g_ vibration mode. The strong peak at 667 cm^−1^ is related to the inverse spinel structure of magnetite [23]. The large line widths (approx. 48 cm^−1^) can be interpreted in terms of the presence of static electronic disorder due to the random arrangement of Fe^2+^ and Fe^3+^ on the B sites and dynamic disorder due to the hopping of polarons from Fe^2+^ to Fe^3+^ sites [24]. Other typical magnetite lines, observed at 534 cm^−1^, 308 cm^−1^ and 191 cm^−1^, are attributed to the three T_2g_ modes of vibration. The T_2g_ modes originate from vibrations involving Fe^3+^ and O^2−^, while Fe^2+^ are not directly involved. There is no additional peak that corresponds to any other phase of iron oxide.

The Raman spectrum of the F2 sample is more complex and contains more lines. Magnetite characteristic bands are identified at 665 cm^−1^, 525 cm^−1^, 311 cm−1, and 191 cm^−1^, similarly to the previous sample (Figure 2). The lines correspond to the A_1g_ and T_2g_ modes. Band observed at 464 cm^−1^ can be assigned to an optical magnon excitation [25,26]. The appearance of additional lines (at about 700 cm^−1^, 380 cm^−1^, 275 cm^−1^) can be associated with non-stoichiometric in the sample or some of its disorder.

**Figure 3:**
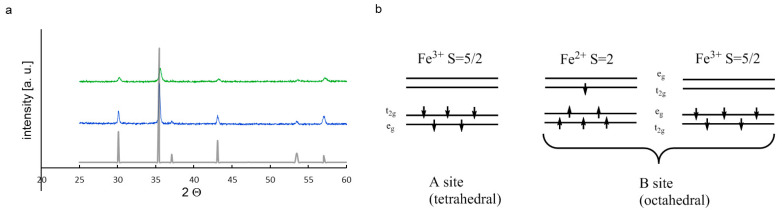
XRD patterns of F1 (blue), F2 (green), reference data code 01-089-3854 (grey) (**a**); Electronic configuration of iron ions in magnetite ([Fe^3+^]_A_[Fe^2+^Fe^3+^]_B_O_4_) (**b**).

XRD (X-ray powder diffraction) measurements show diffraction peaks at 2θ = 30.2°, 35.3°, 43.7°, 53.9°, and 57.1° (Figure 3a). These results are in good agreement with Fe_3_O_4_ reference data (reference code 01-089-3854), attributed to cubic structure system. They correspond to the (220), (311), (400), (422), (511) and (440) facets of Fe_3_O_4_, respectively [27].

The crystallite size of nanoparticles was obtained using the Scherrer equation [28,29]:

D = 0.89λ/βcosϑ(1)

where λ, β and θ are the X-ray wavelength, full width at half maximum (FWHM, in radians) and Bragg angle, respectively. The average crystallite sizes of F1 and F2 catalyst are 46 and 28 nm, respectively. The lengths of the edges of the magnetite unit cells measured for F1 and F2 were 8.3845 Å and 8.3595 Å, respectively [30].

Fe_3_O_4_ has an inverse spinel structure, containing oxygen ions and iron ions that form the tetrahedral and octahedral structures, represented by [Fe^3+^]_A_[Fe^2+^Fe^3+^]_B_O_4_, in which the tetrahedral position is occupied by Fe^3+^ ions and the octahedral position is occupied by eight Fe^2+^ and eight Fe^3+^ ions. Electronic states are mainly threefold t_2g_ and twofold e_g_ from crystal field splitting. The exchange interaction results in high-spin configurations, as shown in Figure 3b, where Fe^3+^ A and B sites can be represented by e_g_^2↓^t_2g_^3↓^, and t_2g_^3↓^e_g_^2↓^, respectively, and Fe^2+^ B sites can be represented by t_2g_^3↑^e_g_^2↑^t_2g_^↓^. Because of the double exchange interaction existing between Fe^2+^ and Fe^3+^ in octahedral sites due to the d orbital overlap between the iron atoms, the additional spin-down electron can hop between neighbouring octahedral sites, resulting in high conductivity at room temperature [31,32].

This electron-hopping process highly depends on the stoichiometry of the magnetite. Magnetite stoichiometry (represented as n(Fe^2+^)/n(Fe^3+^) = x) strongly influences its properties, including magnetic coercivity, sorption capacity, crystal structure, and optical properties [33]. Magnetite crystallites are larger than those of the maghemite, the unit cell edge length is 8.40 Å and 8.34 Å, respectively. Górski et al. [34] showed that the length of the edge of the magnetite unit cell is linearly related to the stoichiometry For x = 0, a = 8.3390 Å; x = 0.25, a = 8.3662 Å; x = 0.5, a = 8.3942 Å). Using these data and the unit cell edge length values obtained from XRD measurements of the materials F1 and F2 (8.3845 and 8.3595 Å, respectively) the x values were determined. The studied catalysts were non-stoichiometric magnetites with the x-value equal to 0.4094 and 0.1828 for F1 and F2, respectively [30]. Therefore, F1 exhibits the properties of a conductor (E_bg_ = 0.11 eV, Figure S1), while F2 is a semiconductor (E_bg_ = 1.75 eV). The electron hopping process has been described as a localised process, limited only to the available Fe^2+^-Fe^3+^ pairs [35], which means that the electron hopping process depends on the degree of non-stoichiometry.

The electrostatic interaction between the catalyst surface and the organic molecule is a very important factor that affects the efficiency of heterogeneous catalysis. The pH_pzc_ of the F1 and F2 powders is due to the surface acid-base properties. This point was at pH = 8 for F1 and at pH = 6.2 for F2 (Figure S2). Close to pH_pzc_ the catalyst surface has a neutral charge (≡Fe(II,III)OH), while under alkaline conditions (pH > pK_a2_), the catalyst surface is deprotonated (Equation (2)), forming ≡Fe(II,III)O^–^ as the dominant species.

(2) $$\equiv \mathrm{Fe}\left(\mathrm{II},\mathrm{III}\right){\mathrm{OH}}_{2}^{+}\overset{H^{+},pK_{1}}{\to }\equiv \mathrm{Fe}(\mathrm{II},\mathrm{III})\mathrm{OH}\overset{{-H}^{+},pK_{2}}{\to }\equiv {\mathrm{Fe}\left(\mathrm{II},\mathrm{III}\right)O}^{-}$$

**Figure 4:**
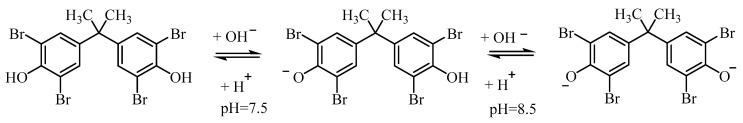
TBBPA ionic equilibrium depending on pH.

That means that the catalyst surface under the experimental conditions was negatively charged (≡Fe(II,III)O^–^). The ionic composition of the TBBPA solution depends on the pH of the solution as shown in Figure 4. Analysing the ionic composition of TBBPA under the reaction conditions, one can see that 87% of the compound is present as a dianion and 13% as a mono anionic compound. The negative charge on the catalyst surface repels negatively charged organic molecules, which reduces the rate of degradation.

Nevertheless, the reaction of TBBPA with a photocatalytically generated electron was observed, suggesting an interaction between the catalyst surface and the TBBPA molecule.

### 3.2. Catalytic Properties

The experiments were carried out under reductive and oxidative conditions, to evaluate the degradation efficiency of these pathways. All experiments were performed at pH = 10. At this pH the high stability of magnetic powders and also the good solubility of TBBPA is expected [30]. It is also worth mentioning that alkaline conditions are suitable for reduction processes [36].

Under the conditions of experiments, the reactions approximate pseudo-first-order kinetics (Equation (3)).

(3) $$ln\frac{C_{t}}{C_{0}}=-k_{app}t$$

where k_app_ is the apparent rate constant; C_0_ and C_t_ are the initial concentration and concentration at time t.

**Figure 5:**
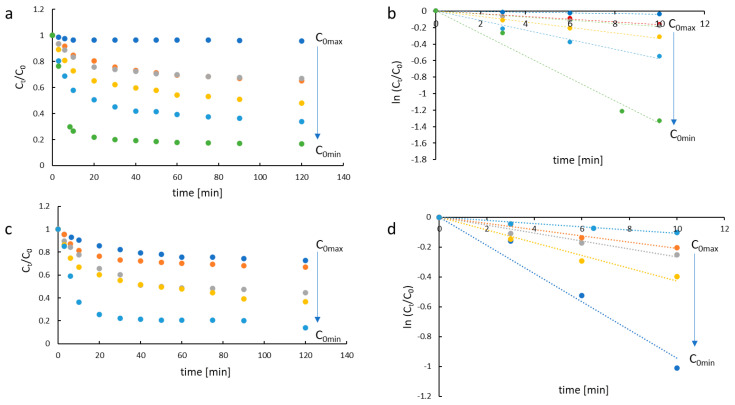
TBBPA decay in time at different initial concentrations on F1 (**a**) and F2 (**c**) catalyst; linear variation of ln(C_t_/C_0_) versus time for the photocatalytic degradation of TBBPA at different initial concentrations on F1 (**b**) and F2 (**d**) catalyst; reductive conditions.

The values of *k_app_* are obtained directly from the linear regression analysis (Figure 5b,d). The values which correspond to different initial concentrations, along with the regression coefficients, are listed in Table 1.

Basically, two different mechanisms are considered to describe photocatalytic processes in heterogeneous systems: (a) the Langmuir-Hinshelwood (LH) mechanism, in which reactants are pre-adsorbed on the photocatalyst surface before photoactivation of the system, and (b) the Eley-Rideal (ER) pathway, when the reagents in the solution react with the active state of the photocatalyst surface [37]. Since, the adsorption of TBBPA on the catalyst surface is strongly influenced by repulsion, the Langmuir-Hinshelwood model cannot be used for the studied system.

**Figure 6:**
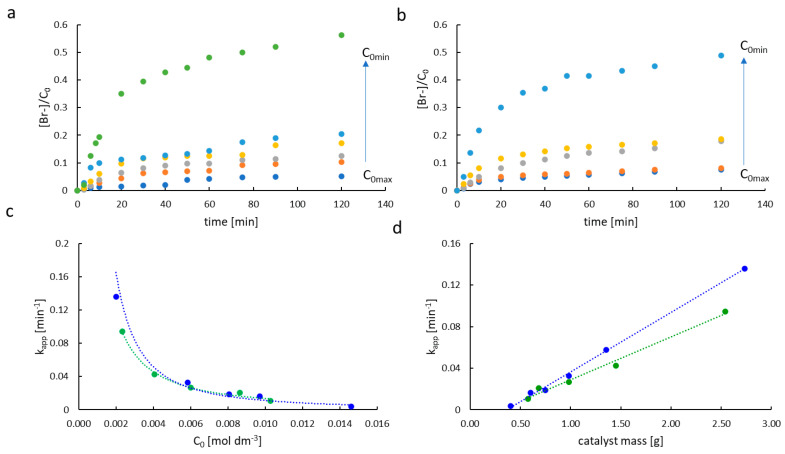
Bromide production in time for different TBBPA concentration on F1 (**a**) and F2 (**b**) catalyst; effect of TBBPA initial concentration on k_app_ for F1 (blue) and F2 (green) catalysts (**c**); apparent rate constant as function of catalyst loading F1—blue, F2—green (**d**); reductive conditions.

For all concentrations, good agreement with the first-order reaction can be observed. The effect of time and initial concentration on the efficiency of the photocatalytic degradation of TBBPA was investigated (Figure 5a,c). It can be seen that the substrates gradually disappear during photocatalysis. Simultaneously, with the disappearance of the substrate, bromide ions appear in the reaction mixture (Figure 6a,b). As the initial concentration of TBBPA decreases, the reaction rate increases (Figure 6c).

The relationship between the rate constant and the amount of catalyst tested for the 10 mM TBBPA solution is linear (Figure 6d). This means that the apparent rate of the TBBPA degradation reaction increases with the increase in the amount of catalyst. The greater the amount of catalyst, the greater the probability of TBBPA-catalyst surface interaction, and thus the probability of the reaction increases. On the other hand, increasing the catalyst content in the reaction mixture may increase the opacity of the suspension, which may lead to a reduction in the amount of light absorbed by the catalyst.

**Figure 7:**
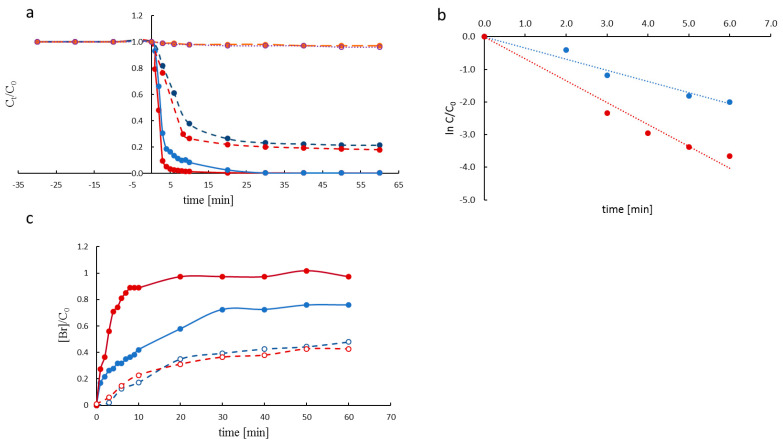
Comparison of TBBPA (0.002 mol dm^−3^) decay in time in reductive (dashed line, F1-blue, F2 red) and oxidative (continuous line, F1—blue, F2—red) processes on catalyst’s surface, control experiment without of light (dotted violet line—F1, dashed orange line—F2) (**a**); linear variation of ln(C_t_/C_0_) versus time for the oxidative degradation of TBBPA (**b**); bromide production in time in oxidative process (filled circles) on F1 (blue) and F2 (red) and reductive process (empty circles, F1-blue, F2-red) (**c**).

The rate of reductive degradation reaches a plateau in about 20 min (80% degradation of TBBPA (Figure 7a), while the oxidation experiment leads to the complete disappearance of TBBPA in about 30 min. The higher effectiveness of the oxidative process is reflected in the k_app_ values, which were 0.3434 and 0.6713 min^−1^ for F1 and F2 (Figure 7b), respectively. The amount of bromides formed in the oxidation process is about twice as high as in the reduction process (Figure 7c) and corresponds to the formation of one bromide anion from one TBBPA molecule. In the oxidative process, the oxygen present in the solution scavenges electrons to form the O_2_^•−^ anion radical (Equation (4)), and ^•^OH radicals are formed (Equation (5)) which can react with TBBPA at the depth of the solution, while electrons can react with TBBPA only on the catalyst surface. Photogenerated holes also contribute to the degradation of TBBPA under oxidative conditions (Equation (7)). The t-BuOH was used as the scavenger of holes, hydroxyl radicals and the hydrogen atoms in the reductive experiment according to Equations (8)–(10). However, due to the pH of the degradation reaction, the formation of a hydrogen atom is negligible. Hence, the lower yield of the TBBPA reaction in the reduction process.

(4) $$O_{2}+e^{-}\to O_{2}^{•-}$$

(5) h++OH−→OH•

(6) $$H^{+}+e^{-}\to H$$

(7) $$h^{+}+{TBBPA}^{2-}\to {TBBPA}^{•-}$$

(8) CH33COH+H→C•H2CH32COH+H2

(9) CH33COH+OH•→C•H2CH32COH+H2O

(10) CH33COH+h+→C•H2CH32COH+H+

The irradiation of the catalyst causes band-gap excitation and generation of valence-band holes and conduction-band electrons. The presence of a hole scavenger (*t*-BuOH) causes an accumulation of electrons on the surface of the catalyst. The electrons can activate a carbon-halogen bond via DET (dissociative electron transfer) to produce a carbon-centered radical and halide anion. Transfer of the surface-trapped electron to the π* orbitals of TBBPA is the rate-limiting step [38]. It is followed by rapid intramolecular electron transfer to the anti-bonding σ* orbital of C-Br, producing aryl radical and bromide ion. It is preferable to cleave one bromide anion from one TBBPA molecule because compounds containing fewer halogen atoms are more resistant to reductive conversion (due to the increase in the electron density of the aryl ring with the decrease of halogen’s substituents number) [39].

**Figure 8:**
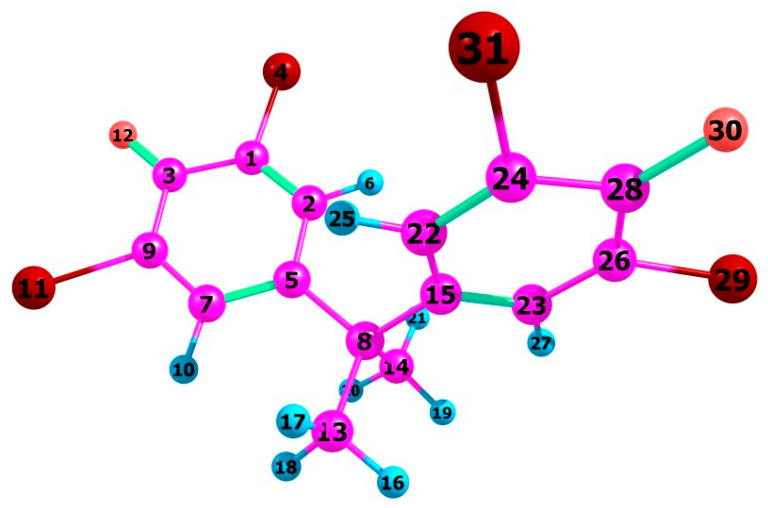
Geometry of reduced form of TBBPA (TBBPA^●−^) calculated using LC-ωPBE 6-311++G(d,p) theory level.

However, LC-ωPBE 6-311++G(d,p) calculation of reduced form of TBBPA (TBBPA^●−^) (Figure 8) shows a relatively stable structure (without debromination). This suggests a two-step reductive mechanism. In the first step, radical anion of TBBPA^●−^ is generated as a consequence of TBBPA reduction by e^−^. In the next step, TBBPA^●−^ realises Br^−^. Relatively stable (long lived) TBBPA^●−^ can undergo other than debromination reaction. Therefore, all e^−^ may not be quantitatively converted to Br^−^. This is in contrast with fast, one-step DET mechanism. On the other hand, it is important to note that the high solvation energy of Br^−^ (303 kJ/mol [40] favours debromination and can be the driving force of the considered reaction. The spatial configurations and charge distribution of TBBPA and TBBPA^●−^ shows that bromine atoms attached to rings of TBBPA are not equivalent. Therefore, the detachment of the Br atom at a certain position can be preferential. From our calculation, the lowest energies were obtained for the debrominated TBBPA radical structure at C-position 1 or 24 (see Figure 8). However, radicals formed by the debromination of TBBPA^●−^ at the position 9 or 26 have only 6.2 kJ/mol higher free Gibbs energy than the previous one. This value is on the thermal (k_b_T) level. Thus, no selectivity is expected in the debromination of TBBPA.

Charges distribution (with hydrogens charges summed into heavy atoms) over TBBPA and TBBPA^●−^ was calculated using LC-ωPBE 6-311++G(d,p) level of theory and visualised by the Mulliken method and shown in Table 2.

Functional groups that donate/receive an electron can also affect the thermodynamics of the reaction by shifting the energy level of unoccupied substrate orbitals [41]. The bond cleavage of anion radical ArX^•−^ competes with the back electron transfer to valence bond hole, which reduces the efficiency of the dehalogenation reaction and degradation rate. The possible degradation pathway in the oxidative process consists of an initial reductive debromination and further oxidation leading to low molecular products. The slightly lower efficiency of the reduction process on the F2 (k_app_ values were 0.1358 and 0.0916 min^−1^ for F1 and F2, respectively) catalyst may be due to the higher degree of oxidation of this material [30], and thus a smaller amount of Fe^2+^ ions at the B sites and the limitation of electron hopping between Fe^3+^ and Fe^2+^ ions at the B sites. The opposite tendency (k_app_ values for F1 and F2 were 0.3434 and 0.6713 min^−1^, respectively) observed in the case of the oxidation process is probably caused by the morphology of the F2 catalyst. It consists of large agglomerates of small crystallites, which cause its porous structure.

When the oxygen is present in the reaction mixture, it works as an electron scavenger and competes with TBBPA for electrons from Fe^2+^ to produce reactive oxygen species including O_2_^•−^ and ^•^OH radicals, as shown in the following reactions:

(11) $${Fe}^{2+}+O_{2}⟶O_{2}^{•-}+{Fe}^{3+}$$

(12) Fe3++OH−⟶Fe2++OH•

**Scheme 1:**
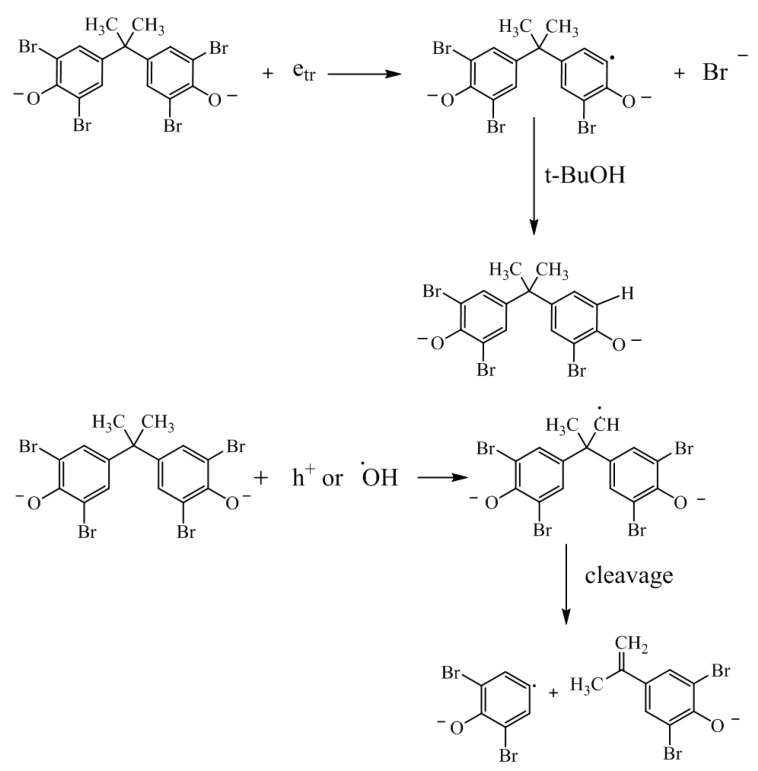
Suggested TBBPA reaction with trapped electron and hole or ^•^OH.

These radicals can react with TBBPA in solution bulk, and result in a higher efficiency of the oxidative degradation reaction. The suggested degradation pathway for TBBPA in reaction with trapped electron (e_tr_) and oxidative species such as ^•^OH and hole (h^+^) are presented on Scheme 1.

The scavenging of electrons by O_2_ enhances the oxidation reaction, involving photo-excited holes and/or hydroxyl radicals, causing cleavage, chemical cleavage, or ring opening to produce low molecular weight products. By-products were qualitatively estimated by GC-MS analysis (Figure S4). The reaction mixture (oxidative conditions) contained following degradation products: BPA, 2,6-dibromo-4-isopropylphenol, and other aromatic compounds (e.g., 3,5-dibromo-4-hydroxybenzoic acid, 2,6-dibromo-4-methylphenol, bromophenol, phenol).

## 4. Summary

Fe_3_O_4_ was studied as a heterogeneous catalyst for the degradation of polyhalogenated water pollutants. Results showed that magnetite could effectively catalyse TBBPA degradation under reductive and oxidative conditions. Under all experimental conditions, the reactions followed pseudo-first-order kinetics. Within 30 min, approximately 80% and 99% of TBBPA were degraded under reductive and oxidative conditions, respectively. The degradation reaction of TBBPA under reducing conditions proceeds with a photogenerated surface electron. Due to the catalyst surface properties and weak adsorption of TBBPA on the catalyst surface, the photocatalytic degradation reaction follows the Eley-Rideal mechanism. The ^•^OH radicals generated by the reaction of ^−^OH anion (or H_2_O molecule) with photogenerated hole and O_2_^•−^ anion radical created by the reaction of molecular oxygen with photogenerated electron are the main radicals involved in TBBPA degradation under oxidative conditions.

The electron transfer process plays a significant role in photocatalysis, hence the electron hopping in the Fe^2+^-Fe^3+^ pair on the catalyst surface handled the formation of radicals.

The obtained results suggest the efficiency of the electron transfer process and hence the overall photocatalytic process depends on the photocatalyst size, surface properties, morphology and electronic configurations of atoms.

## Figures and Tables

**Table 1:** Pseudo-first order apparent constant values for the different initial concentrations of TBBPA (reductive conditions).

C_0_ [mol dm^−3^]	k_app_ [min^−1^]	R^2^
F1		
0.01462	0.0037	0.99
0.00985	0.0164	0.97
0.00790	0.0187	0.99
0.00602	0.0328	0.99
0.00437	0.0578	0.98
0.00216	0.1358	0.98
F2		
0.0103	0.0108	0.99
0.0086	0.0208	0.99
0.0060	0.0266	0.99
0.0041	0.0426	0.99
0.0023	0.0945	0.98

**Table 2:** Mulliken charges with hydrogens summed into heavy atoms calculated for TBBPA and TBBPA^●−^ calculated using LC-ωPBE 6-311++G(d,p) theory level.

Atom Number	Atom Type	Charges on TBBPA	Charges on TBBPA^●−^
1	C	0.3645	0.1697
2	C	−0.6360	−0.8545
3	C	−0.8366	−0.5303
4	Br	−0.2694	−0.3020
5	C	0.8157	−0.3380
7	C	−0.2371	0.8684
8	C	0.3232	−3.1199
9	C	0.5651	1.8624
11	Br	−0.2802	−0.4522
12	O	−0.3898	−0.4147
13	C	−0.2579	0.0512
14	C	−0.2579	0.0512
15	C	0.8158	−0.3379
22	C	−0.6360	−0.8545
23	C	−0.2371	0.8683
24	C	0.3644	0.1697
26	C	0.5650	1.8623
28	C	−0.8365	−0.5303
29	Br	−0.2802	−0.4522
30	O	−0.3900	−0.4147
31	Br	−0.2694	−0.3020

## Data Availability

The data presented in this study are available on request from the corresponding author. The data are not publicly available because they are part of the ongoing project.

## Supplementary Materials

The following supporting information can be downloaded at: https://www.mdpi.com/article/10.3390/ma16124380/s1, Figure S1: Tauc plots of F1 (blue); F2 (green); Figure S2: pH changes of 0.1 M NaCl solutions in the presence of F1 (blue) and F2 (green); Figure S3: Hydrodynamic diameter of F1 (blue), F2 (green) in water suspension; Figure S4: GC-MS analysis of reaction mixture, where a—TBBPA; b—BPA; c—3,5-dibromo-4-hydroxybenzoic acid; d—2,6-dibromo-4-isopropylphenol; e—2,6-dibromo-4-methylphenol; f—aliphatic carboxylic acids; g—tri-, di-, mono-bromobisphenol A; h—phenol.
