# Supplementary material for: Reductive Photodegradation of 4,4′-Isopropylidenebis(2,6-dibromophenol) on Fe3O4 Surface
Source: Materials (Basel). 2023 Jun 14;16(12):4380. doi: 10.3390/ma16124380 (PMC10303975; doi:10.3390/ma16124380)
Supplement: Supplementary file 1 [file materials-16-04380-s001.zip › materials-2400490-supplementary.pdf]

Supplementary Materials:

Reductive photodegradation of 4,4'-isopropylidenebis(2,6-dibromophenol) on Fe_3O_4 surface

Joanna Kisała [1,*], Bogdan Stefan Vasile [2], Anton Ficai [3], Denisa Ficai [3], Renata Wojnarowska-Nowak [4], Tomasz Szreder [5]

[1] Institute of Biology, College of Natural Sciences, University of Rzeszow, Pigonia 1 Str., 35-310 Rzeszow, Poland; jkisala@ur.edu.pl
[2] University POLITEHNICA of Bucharest; Splaiul Independentei 313, Bucharest, Romania; bogdan.vasile@upb.ro
[3] Faculty of Chemical Engineering and Biotechnologies, University POLITEHNICA of Bucharest, 1-7 Gh. Polizu st. Bucharest, Romania; anton.ficai@upb.ro, denisa.ficai@upb.ro
[4] Institute of Materials Science, College of Natural Sciences, University of Rzeszow, Pigonia 1 Str., 35-959 Rzeszow, Poland; rwojnarowska@ur.edu.pl
[5] Institute of Nuclear Chemistry and Technology, Dorodna 16, 03-195, Warsaw, Poland; t.szreder@ichtj.waw.pl
* Correspondence: jkisala@ur.edu.pl; Tel.: +488518581

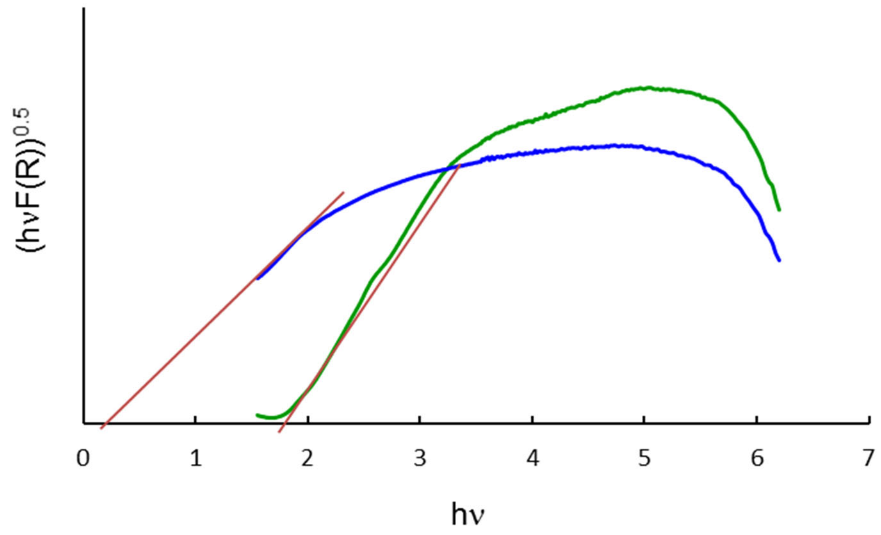

Figure S1. Tauc plots of F1 (blue); F2 (green).

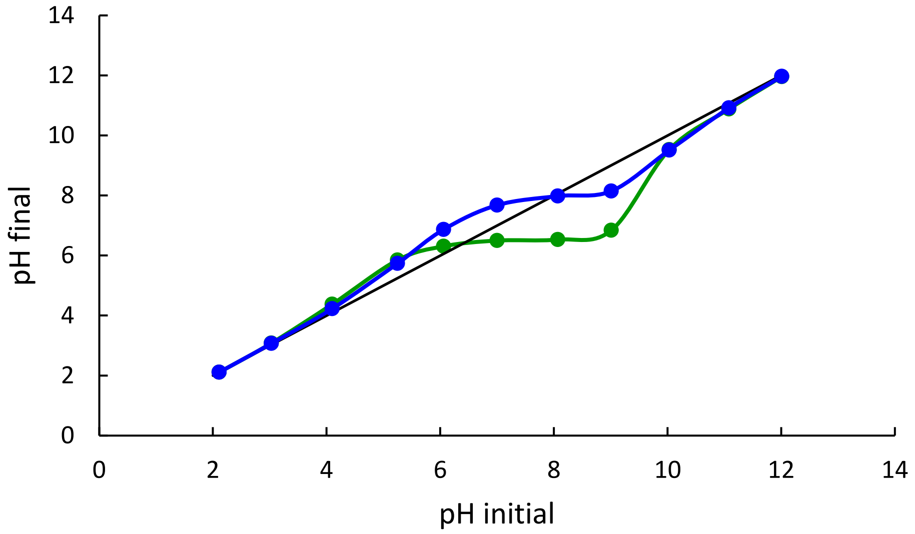

Figure S2. pH changes of 0.1 M NaCl solutions in the presence of F1 (blue) and F2 (green).

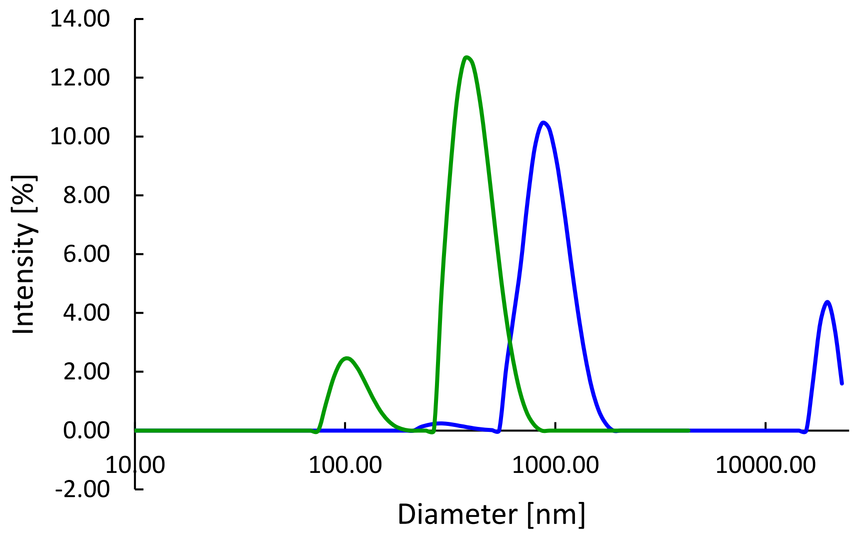

Figure S3. Hydrodynamic diameter of F1 (blue), F2 (green) in water suspension.

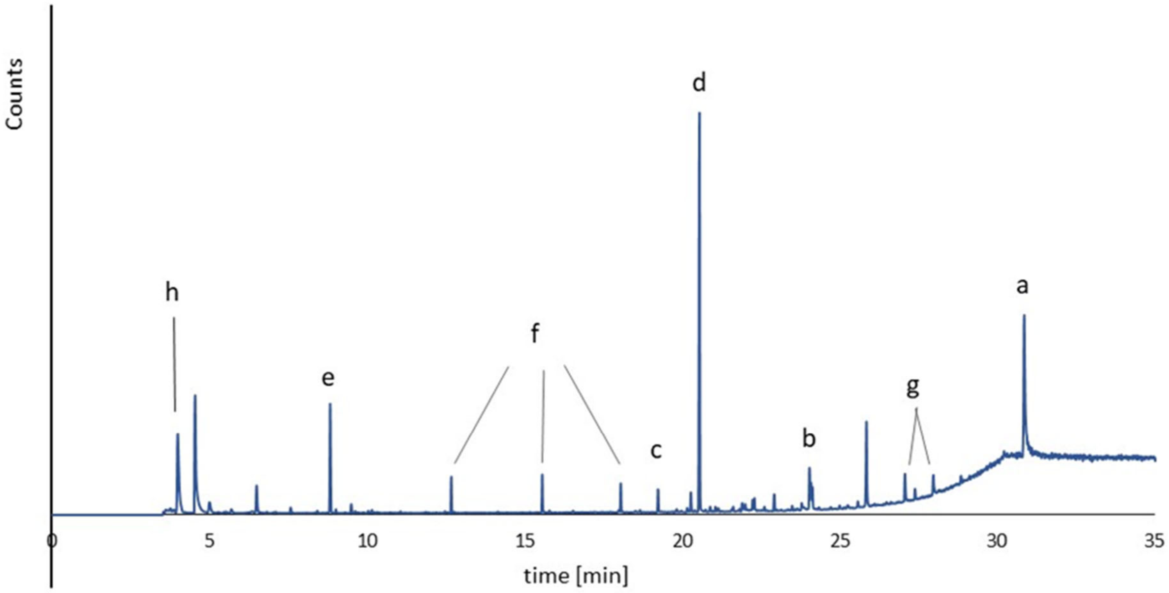

Figure S4. GC-MS analysis of reaction mixture, where a – TBBPA; b – BPA; c - 3,5-dibromo-4-hydroxybenzoic acid; d - 2,6-dibromo-4-isopropylphenol; e - 2,6-dibromo-4-methylphenol; f – aliphatic carboxylic acids; g – tri-, di-, mono-bromobisphenol A; h – phenol.
